# Building predictive Markov models of ion channel permeation from molecular dynamics simulations

**DOI:** 10.1016/j.bpj.2024.09.030

**Published:** 2024-09-28

**Authors:** Luigi Catacuzzeno, Maria Vittoria Leonardi, Fabio Franciolini, Carmen Domene, Antonio Michelucci, Simone Furini

**Affiliations:** 1 Department of Chemistry, Biology and Biotechnology, University of Perugia, Italy; 2 Department of Chemistry, University of Bath, Bath, United Kingdom; 3 Department of Electrical, Electronic and Information Engineering “Guglielmo Marconi”, University of Bologna, via dell’Università 50, Cesena (FC), Italy

## Abstract

Molecular dynamics (MD) simulation of biological processes has always been a challenging task due to the long timescales of the processes involved and the large amount of output data to handle. Markov state models (MSMs) have been introduced as a powerful tool in this area of research, as they provide a mechanistically comprehensible synthesis of the large amount of MD data and, at the same time, can be used to rapidly estimate experimental properties of biological processes. Herein, we propose a method for building MSMs of ion channel permeation from MD trajectories, which directly evaluates the current flowing through the channel from the model’s transition matrix (*T*), which is crucial for comparing simulations and experimental data. This is achieved by including in the model a flux matrix that summarizes information on the charge moving across the channel between each pair of states of the MSM and can be used in conjunction with *T* to predict the ion current. A procedure to drastically reduce the number of states in the MSM while preserving the estimated ion current is also proposed. Applying the method to the KcsA channel returned an MSM with five states with significant equilibrium occupancy, capable of accurately reproducing the single-channel ion current from microsecond MD trajectories.

## Significance

The high efficiency of permeation through ion channels is still not fully understood despite the knowledge of numerous ion channel atomic structures. This paper proposes an analysis of molecular dynamics simulations of permeation through ion channels, which allows the permeation process to be described in terms of a few relevant selectivity filter ion configurations connected to form a kinetic scheme. The same analysis returns transition rate constants connecting the various states that are numerically exact and able to predict the correct ion current. The kinetic model resulting from the application of the proposed method may help to understand the experimental data on ion channel permeation and the structural significance of the kinetic models used in the past to explain the permeation process.

## Introduction

Ion channels are membrane proteins that allow the passage of ions across cell membranes, remarkably combining high-throughput rate and high selectivity, and this combination of properties has interrogated scientists for decades. A turning point for the understanding of these astonishing features has been the possibility to obtain high-resolution atomic structures of ion channels, fundamental to relate permeation and selectivity processes to chemical structures forming the ion channel pores (1). Among the advantages offered by the knowledge of ion channel structures, there is the possibility to investigate the dynamic mechanisms of ion conduction at the atom scale by molecular dynamics (MD) simulations (2,3).

All-atom MD simulations, which define the state of the system by considering the coordinates and velocities of all atoms, consist of a numerical integration of the classical equation of motion, combined with algorithms for sampling configurations in the proper thermodynamic ensemble. Because of this complexity, microsecond trajectories—a time window that allows the observation of only a scanty number of ions crossing the channel at physiological conditions—require weeks of computation in ordinary workstations. This limited amount of information is not sufficient to study and understand mechanisms such as permeation and selectivity. A second major shortcoming of MD simulations is the overwhelming amount of data to process (i.e., the positions and the velocities of all the atoms over time), most of which are irrelevant for understanding the processes of interest. This massive amount of information might hinder the identification of forces and structures relevant for the process under investigation. Given these limitations, other strategies should be considered to complement MD simulations for studying permeation in ion channels. One promising approach is to combine MD simulations with Markov state models (MSMs).

MSMs are a powerful strategy for extracting relevant information from MD trajectories in a statistically robust way (4). The analysis of MD trajectories using MSMs entails the following steps (5): 1) the definition of a preliminary set of input features, 2) the clustering of the trajectories using these input features to define the microstates of the system, 3) the calculation of the transition matrix (*T*) among these microstates, and 4) the coarse graining of the estimated MSM into a limited number of long-lived metastable states. These metastable states group together microscopic states that rapidly interconvert into each other while separating microscopic states that are distant in time, offering in this way a simplified description that captures the most important dynamic events in the system. Previous applications of MSMs to the analysis of permeation and selectivity in ion channels have been reported by Harrigan et al. (6), Furini and Domene (7), Domene et al. (8), and Lam and de Groot (9). These studies represent a proof of principle of how MSMs can be useful for the analysis of ion permeation, but they do not provide a formalism to predict the ion channel current directly from the *T* of the MSM, nor to coarse grain the MSM to few relevant macrostates while preserving the capacity to correctly predict the ion current.

**Figure 1:**
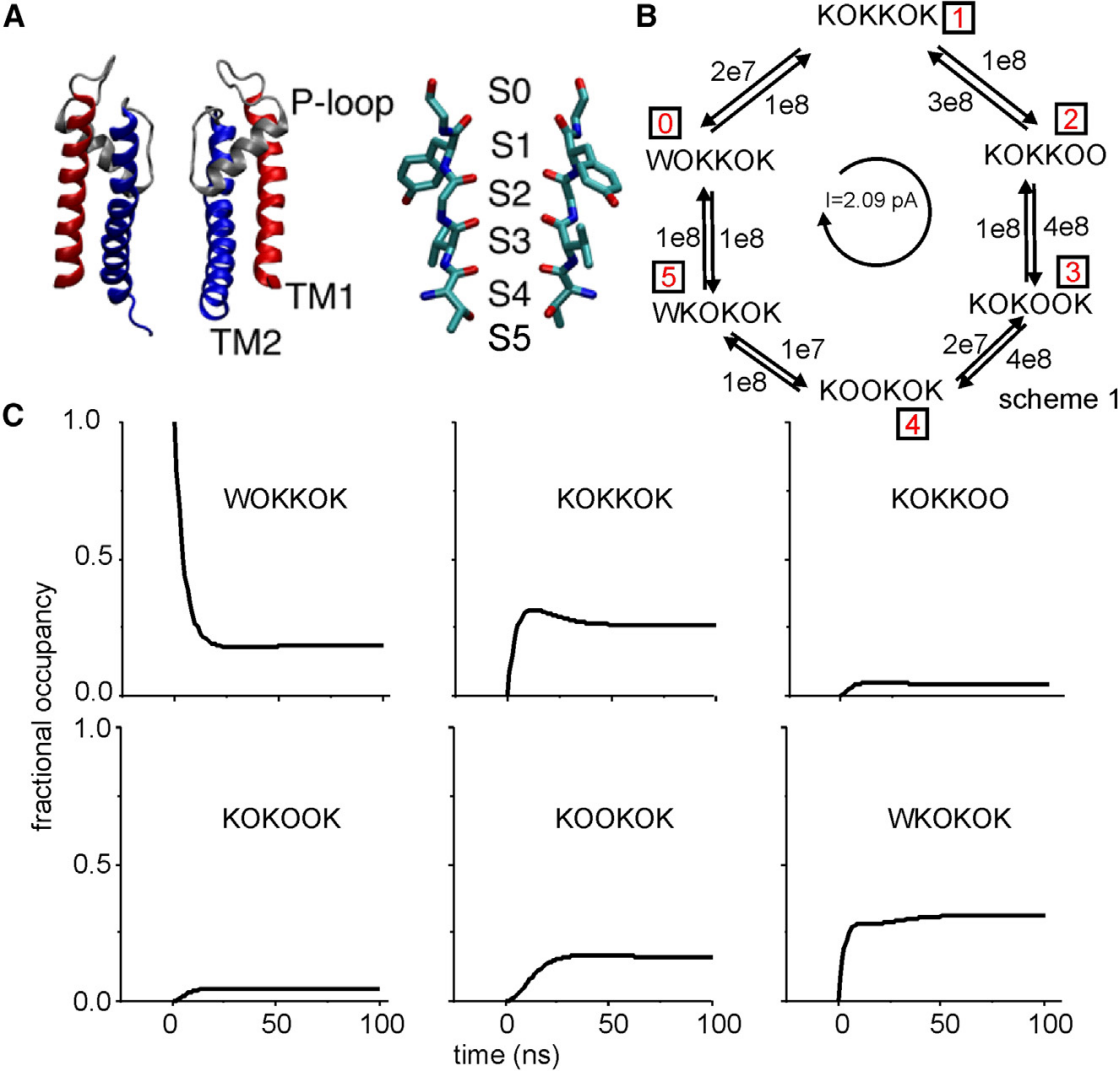
(*A*) Atomic structure of the KcsA channel (*left*, only two of the four subunits are shown) and its SF (*right*), showing the six binding sites (S5-S0). (*B*) Exemplificative six-state MSM considered in the text. States 0 to 5 correspond to different occupancy states of the SF. Numbers represent rate constants (in s^−1^). (*C*) Prediction of the occupancy of the six states of the scheme in (*B*) versus time (Eq. 3), starting from an initial condition in which the probability occupancy of state 0 (WOKKOK) is unitary.

The purpose of this study is to build a method that correctly recovers from MD data an MSM of ion permeation, defined by a limited number of discrete states and the corresponding transition rates among them, which can be used to predict the ion current. Such an MSM offers an immediate understanding of the system dynamics. The method was tested using MD data of the bacterial KcsA K channel (10). This channel has similar structural properties to many eukaryotic K channels, making it suitable for the understanding of K transport (11,12). KcsA is formed by the juxtaposition of four identical subunits, each composed of two transmembrane segments, TM1 and TM2, that make up the permeation pore (Fig. 1 *A*, *left*). At the pore extracellular entrance, the four P-loops connecting the TM1 and TM2 segments of each subunit contribute to the narrow selectivity filter (SF), about 12 Å long and 3 Å wide, formed by a highly conserved sequence of amino acids (TVGYG in most K channels), with their carbonyl oxygen atoms pointing toward the pore. Together with the hydroxyl oxygens of the threonine- residues, these carbonyl oxygens define a series of binding sites for K, named S4 to S1, from the intracellular side (Fig. 1 *A*, *right*). Two additional binding sites, immediately at the intracellular and extracellular entrances of the SF, called S5 and S0, respectively, were also identified (12,13,14). We performed extensive MD simulations obtaining tens of K ion passages through the SF. We then used these data to build a reduced MSM containing only five relevant states and capable of reproducing an ion current comparable to that obtained by directly counting the ion passage events from MD data (see Fig. 8).

## Materials and methods

### MD simulations

The channel model was based on the experimental structure of KcsA-E71A in the open/conductive state, PDB: 5VK6 (15). The entire transmembrane domain of the channel, from residue Trp26 to residue Gln121, was considered. The initial atomic coordinates of the systems were obtained with CHARMM-GUI (16). The lipid membrane was a mixture of 1-palmitoyl-2-oleoyl-glycero-3-phosphocholine and 1-palmitoyl-2-oleoyl-sn-glycero-3-phosphate, with a ratio of 3:1. The channel was inserted in the lipid membrane as defined in the Orientations of Proteins in Membranes database (17). The system was solvated using TIP3P water molecules (∼15,000 molecules) and ions to reach 200 mM KCl. Potassium ions were manually placed at binding sites S0, S2, and S4. The ff14sb version was used (18), in combination with ion parameters by Joung and Cheatham (19) for the TIP3P water model (20). van der Waals interactions were truncated at 9 Å. A standard AMBER scaling of 1–4 interactions was applied. The equilibration protocol consisted of 10,000 energy minimizations, followed by 10 ns in the NPT ensemble with timesteps equal to 1 fs and 60 ns in the NPT ensemble with a timestep equal to 2 fs. During the equilibration protocol, restraints on protein and lipid atoms were gradually reduced to zero. Long-range electrostatic interactions were calculated with the particle mesh Ewald method using a grid spacing of 1.0 Å (21). The SETTLE algorithm was used to restrain bonds with hydrogen atoms (22). The temperature was controlled at 310 K by coupling to a Langevin thermostat with a damping coefficient of 1 ps^−1^. A pressure of 1 atm was maintained by coupling the system to a Nose-Hoover Langevin piston, with a damping constant of 25 ps and a period of 50 ps (23). The presence of membrane potentials was mimicked by applying a constant electric field acting in the direction perpendicular to the lipid membrane. The simulations with the external electric field were performed in the NVT ensemble. NAMD2.11 was used for all the simulations (24).

### Transition matrix from MD trajectories

Trajectories were analyzed using the python library MD analysis (25) and the SciPy ecosystem (26). Visual MD was used to inspect trajectories and generate images of the systems (27). The center of mass of the following set of atoms was considered as boundaries for the different channel regions: residue A107; hydroxyl oxygen atom of residue T75; and backbone oxygen atoms of residues T75, V76, G77, Y78, and G79. Ions were considered in binding sites S4-S0 or in the S5 when in between the boundaries of the corresponding binding site and when within 4 Å from the channel axis (8 Å for ions in S5). Using these binding site definitions, each frame of the simulated trajectories was converted into a string describing the occupancy for a posteriori classification. The occupancy state of the channel in every frame of each trajectory was case coded using the following regular expression: [KW][KWO][KWO][KWO][KWOB][KWOB]. The first character of the string was K when a potassium ion was in S5 or W if S5 was only filled with water molecules. Similarly, the next character of the string was K if S4 was occupied by an ion, W if it was occupied by a water molecule, and O if S4 was vacant. To define the state of binding sites S3 to S0, the same criteria were imposed. At times, due to the intrinsic structural nature of S0 and S1, an ion and a water molecule can coexist at these sites, and thus, in this case, the character B was used. Simulated trajectories were converted into sequences of discrete states using the classification of the occupancy states defined above. Transition matrices of the MSMs were estimated by counting state-to-state transitions in these discrete trajectories with the sliding method.

### Theory

#### MSM formalism for ion channel permeation

MSMs have been used for more than 50 years to describe ion permeation through biological ion channels (28,29), long before the knowledge of their atomic structure. In all these models, a certain number of configuration states are identified, each one representing a different arrangement of permeating ions bound to the different binding sites along the permeation pathway (i.e., the channel pore). The tendency of the system to transit from a configuration state, *i*, to another state, *j*, is defined either in terms of a kinetic rate constant, *Q*_*ij*_, or in terms of the probability that transition *i* → *j* occurs in a certain time interval or lagtime *Δt*, *T*_*ij*_(*Δt*). In a homogeneous MSM, the probability of going from state *i* to state *j* only depends on the lagtime, not on the absolute time. *Q*_*ij*_ and *T*_*ij*_(*Δt*) are related to each other by the following relationship:

(1) $$Q_{ij}=\lim_{\Delta t\to 0}\frac{{\left[T-I\right]}_{ij}\left(\Delta t\right)}{\Delta t}\text{,}$$

where *I* is the identity matrix and *T* is the transition matrix containing the *T*_*ij*_(*Δt*) elements, as shown in section 1 of the supporting material. States and transition rates are usually sketched with kinetic schemes (as used in chemical reactions), with arrows between the states indicating possible transitions and numbers associated with each arrow indicating the numerical value of the corresponding rate constant. For exemplificative purposes, we will consider a generic MSM of permeation, characterized by the kinetic scheme in Fig. 1 *B*. The scheme is inspired by the prokaryotic KcsA channel (Fig. 1 *A*). Accordingly, the model considers six adjacent K binding sites along the channel pore (named S5 to S0, going from the intracellular to the extracellular side of the pore). At any given time, the occupancy state of each site was classified as empty (indicated by “O”), bound to a K ion (“K”), bound to a water molecule (“W”), or occupied simultaneously by a water molecule and a K ion (“B”). In the simple example of Fig. 1 *B*, only six of these states were considered.

##### Q and T matrices of MSMs

The element *Q*_*ij*_, with i≠j, of the *Q* matrix represents the kinetic rate constant going from state *i* to state *j*, while the diagonal elements *Q*_*ii*_ are defined as $Q_{ii}=-\sum_{j\neq i}Q_{ij}$. For the scheme of Fig. 1 *B*, the *Q* matrix was assumed to be equal to (in s^−1^):

$$Q=\left[\begin{array}{cccccc} -2e^{8} & 1e^{8} & 0 & 0 & 0 & 1e^{8}\\ 2e^{7} & -1.2e^{8} & 1e^{8} & 0 & 0 & 0\\ 0 & 3e^{8} & -7e^{8} & 4e^{8} & 0 & 0\\ 0 & 0 & 1e^{8} & -5e^{8} & 4e^{8} & 0\\ 0 & 0 & 0 & 2e^{7} & -1.2e^{8} & 1e^{8}\\ 1e^{8} & 0 & 0 & 0 & 1e^{7} & -1.1e^{8} \end{array}\right]\text{,}$$

where rows (columns) 0 to 5 correspond, respectively, to states WOKKOK, KOKKOK, KOKKOO, KOKOOK, KOOKOK, and WKOKOK. Similarly, the transition probabilities for a certain lagtime (*Δt*) can be represented as a transition probability matrix, *T*(*Δt*), where each element *T*_*ij*_(*Δt*) represents the probability of the system going from state *i* to state *j* in a time *Δt* given that the system was in state *i* at time zero. In the *T*(*Δt*) matrix, the diagonal elements are the probabilities that no transition occurs in the lagtime *Δt* so that $\sum_{j=0}^{N-1}T_{ij}\left(\Delta t\right)=1$ for all possible *i*, where *N* is the total number of states (six in the example). It can be proved (see section 1 in the supporting material) that for homogeneous MSMs, the *T* matrix is related to the *Q* matrix by the following relationship:

(2) T(Δt)=Exp(QΔt),

where Exp represents the matrix exponential operator.

The *T* at a certain lagtime *Δt* for an MSM can be used to predict the occupancy of the various states of the model at successive times. More specifically, if the fractional occupancy of the system at time *t* is represented by the row vector *P*(*t*) = [*p*_0_(*t*) *p*_1_(*t*) _… ….._ *p*_*N*-1_(*t*)], where *p*_*i*_(*t*) represents the probability of finding the system in state *i*, then

(3) $$P\left(t+\Delta t\right)=P\left(t\right)\;T\left(\Delta t\right)=P\left(0\right)\;{\left[T\left(\Delta t\right)\right]}^{n}\text{,}$$

where *t* is chosen so that *t* + Δt = *n* Δt. A similar equation may also be written for the time evolution of the *T*:

(4) $$T\left(t+\Delta t\right)=T\left(t\right)\;T\left(\Delta t\right)={\left[T\left(\Delta t\right)\right]}^{n}\text{.}$$

The above equations, known as different forms of the Chapman-Kolmogorov equation, clarify a fundamental property of homogeneous MSMs: the occupancy of the system at a certain time only depends on the status of the system at the previous time step, not on its older history. Fig. 1 *C* shows the evolution of the occupancies of the six states of our illustrative MSM, predicted by Eq. 3, considering a lagtime of 1 ns and assuming that the occupancy of state 0 (WOKKOK) is one at time zero.

The *T* of an MSM has the useful property that if an eigen decomposition of its transpose is performed, then the eigenvector corresponding to the unitary eigenvalue (always present) gives the equilibrium fractional occupancy of the various states, expressed as a row vector, $P_{\inf}$. For our illustrative MSM, the described operation gives

$$P_{\inf}=\left[\begin{array}{cccccc} 0.18 & 0.26 & 0.04 & 0.04 & 0.16 & 0.32 \end{array}\right]\text{.}$$

Fig. 1*C* shows that the equilibrium condition is reached after about 50 ns.

##### The flux matrix of MSMs

**Figure 2:**
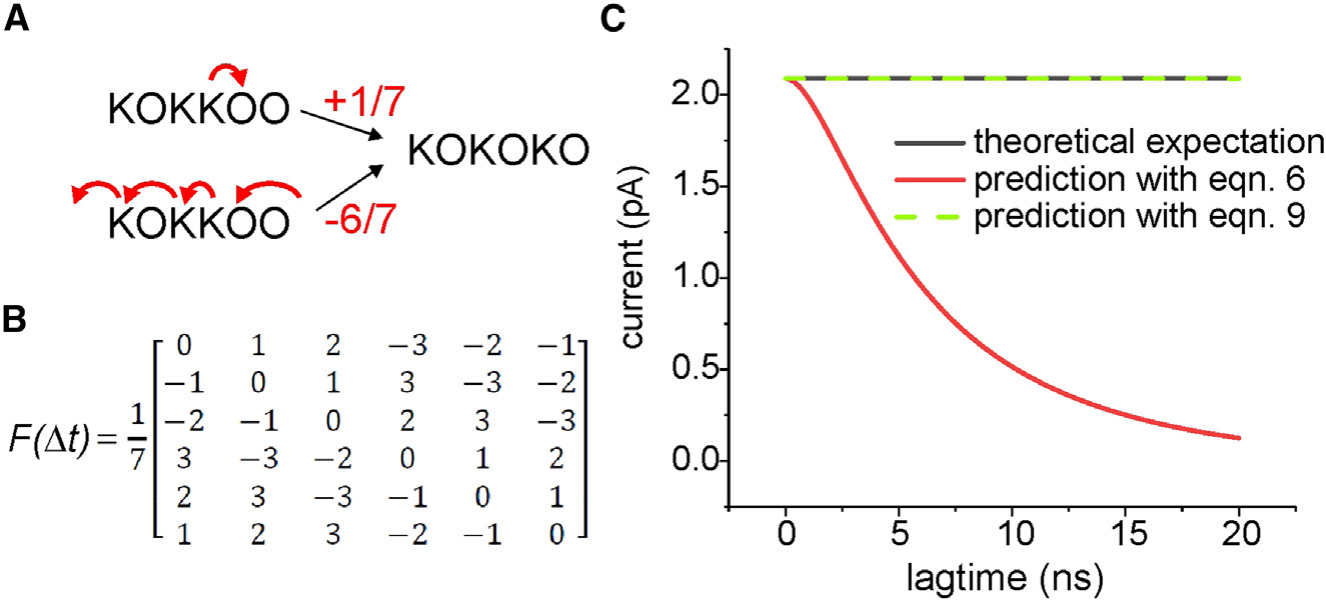
(*A*) Amount of charge translocated according to the route taken to make the transition. (*B*) Flux matrix for the scheme in Fig. 1*B*. (*C*) Ion current prediction at different lagtimes for the six-state MSM using Eqs. 5a and 5b (*black*), 6 (*red*), and 9 (*green*). Note that the theoretical expectation (*black*) superimposes exactly with the Eq. 9 prediction (*green*).

The kinetic scheme of an ion permeation model does not fully define its behavior. To predict the ion current, that is, the ion channel permeation observable during experimental recordings, it is necessary to also know the amount of charge transferred through the permeation pore during each transition. Assuming that the movement of a K ion from a site to the next one immediately to the right (thus, toward the outside; Fig. 2 *A*) has a +1/7 value (if the complete passage of a K from the internal to the external solution has a value of +1), the *F*(*Δt*) for our MSM example, that is, the matrix reporting the charge flux associated to each possible transition, corresponds to the one shown in Fig. 2 *B*.

*F*(*Δt*) can be computed automatically using the algorithm reported in section 2 of the supporting material. Notice that there are, in general, multiple possibilities in going from *i* to *j* and that the algorithm in the supporting material selects the pathway that requires the minimum number of transitions. For example, the transition KOKKOO → KOKOKO has an associated flux of +1/7, obtained by moving the K in S2 to S1. It is, however, possible to reach the same final configuration by moving K ions in the reverse direction, letting the K ion in S5 exit from the pore toward the internal solution, moving the K ion from S3 to S5, the K ion from S2 to S3, and an outside K ion to S1, giving an overall flux of −6/7 (Fig. 2 *A*). The first route is the one selected to define the corresponding element of the *F*, as it is obtained only in one step instead of the six steps needed for the second route.

##### Assessing ion currents from MSMs

There are several ways to assess the ion current (*I*) produced at steady state by an MSM of ion permeation. The most straightforward is to calculate the net rate of entrance (exit) of a K ion from the inside (to the outside). For our exemplificative scheme of Fig. 1 *B*, this would give

(5a) $$I=e_{0}\;\left(P_{\inf,0}\;Q_{01}-P_{\inf,1}\;Q_{10}\right)=e_{0}\;\left(P_{\inf,1}\;Q_{12}-P_{\inf,2}\;Q_{21}\right)\text{,}$$

where $e_{0}$ represents the elementary charge carried by a K ion. A more general expression, working also in more complicated MSMs with multiple points of entrance/exit of ions and using both the *Q* and the *F*, is the following:

(5b) $$I=e_{0}\sum_{i,j=0}^{N-1}P_{\inf,i}\;Q_{ij}F_{ij}\left(\Delta t\right)\text{,}$$

In our example, both the above forms of Eqs. 5a and 5b give a current of 2.09 pA. Equation 6 is an alternative to Eq. 5b, obtained considering discrete time and *T* instead of *Q*:

(6) $$I≅\frac{e_{0}}{\Delta t}\sum_{i,j=0}^{N-1}P_{\inf,i}\;T_{ij}\left(\Delta t\right)\;F_{ij}\left(\Delta t\right)\text{.}$$

As shown by the red line in Fig. 2 *C*, the current estimated by Eq. 6 depends greatly on the lagtime, predicting the correct current at infinitesimal short lagtimes but departing markedly from the theoretical expectation at larger lagtimes. The reason for missing the correct current when using *T*s obtained at large lagtimes originates from the fact that, of the many possible pathways, the *F*(*Δt*) assessed with the algorithm presented in section 2 of the supporting material considers the charge carried following the pathway with the minimum number of steps. While this approximation may be valid for very short lagtimes, increasing the sampling interval, multistep pathways might become more and more likely, and therefore it is necessary to consider these alternatives for a correct prediction. This means that, in general, we need to use a composite *F* that considers the charge carried along all possible pathways that might be followed. More specifically, from Eq. 4, the transition probability from state *i* to state *j* may be written as

(7) $$T_{ij}\left(n\Delta t\right)=\sum_{m=0}^{N-1}{\left[T{\left(\Delta t\right)}^{n-1}\right]}_{im}^{}*T_{mj}\left(\Delta t\right)$$

that decomposes the probability of going from state *i* to state *j* into the different pathways that can be followed. Using the above property, we can assess the composite *F* at a lagtime of *nΔt* using the following equation:

(8) $$F_{ij}\left(n\Delta t\right)=\frac{\sum_{m=0}^{N-1}\left[{\left[T{\left(\Delta t\right)}^{n-1}\right]}_{im}^{}*T_{mj}\left(\Delta t\right)*\left[F_{im}\left(\left(n-1\right)\Delta t\right)+F_{mj}\left(\Delta t\right)\right]\right]}{\sum_{m=0}^{N-1}\left[{\left[T{\left(\Delta t\right)}^{n-1}\right]}_{im}^{}*T_{mj}\left(\Delta t\right)\right]}$$

starting from *F*(*Δt*). Section 3 of the supporting material reports a schematic representation of the assumptions leading to Eq. 8. The *F* obtained in this way provides the average charge carried along with each transition, obtained by a probability-weighted average of all possible pathways, and can be used to predict the current in association with the *T* obtained for the same lagtime using the following relationship:

(9) $$I=\frac{e_{0}}{n\Delta t}\sum_{i,j=0}^{N-1}P_{\inf,i}\;T_{ij}\left(n\Delta t\right)\;F_{ij}\left(n\Delta t\right)\text{.}$$

The green line in Fig. 2 *C* shows that Eqs. 8 and 9 predict the correct value of the ion current using *T*s obtained at any lagtime.

In conclusion, a homogeneous MSM of ion permeation is fully defined by its *T* obtained at a relatively short lagtime, *T*(*Δt*), and the corresponding *F*(*Δt*). With these two pieces of information, we can accurately predict the ion current, provided that the lagtime is sufficiently small. A correct prediction of the ion current can also be recovered using *T*s obtained at larger lagtimes, *nΔt*, provided we find a corresponding composite *F*, *F*(*nΔt*), that considers the average charge flux during *nΔt*.

#### MSM reduction and ion current assessment from the transition probability matrix

A distinctive feature of MSMs built from MD data is the large number of metastable states that usually need to be considered. Obviously, to have an MSM that can help to understand the mechanism of ion permeation, it may be necessary to considerably reduce the number of possible states, using reasonable reduction strategies that lump states together.

##### MSM reduction strategy

**Figure 3:**
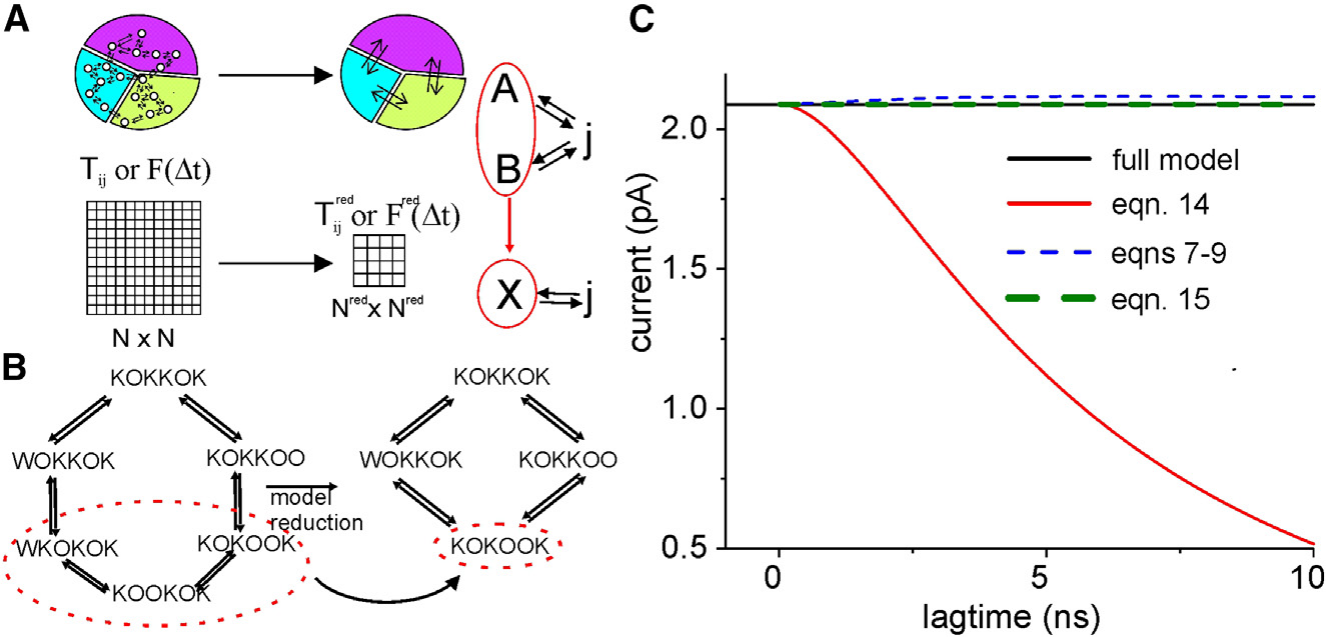
(*A*) Scheme representing the process of reduction of an MSM, considered for both *T* and *F*, so as to have a correct prediction of the ion current. (*B*) Scheme showing the reduction considered for the six-state MSM taken as an example. (*C*) Ion current predicted for the reduced model using Eq. 14 (*red line*), compared with the correct current of the full MSM (*black line*). The plot also shows the current recovered from *T*s at varying lagtimes, using the correction method we proposed for large lagtimes, assuming Markovianity (*dashed blue line*) and assessing the *T* at the various lagtimes without assuming Markovianity (*dashed green line*).

We next illustrate a possible strategy for MSM reduction that preserves steady-state occupancy and flux rates across the different states. We start by considering the process of lumping two states of the model, *A* and *B*, into a single state *X* (Fig. 3 *A*). To preserve the outgoing probability flux and have an identical equilibrium occupancy of the remaining states of the model, we impose that

$$P_{\inf,A}\;T_{A,j}+P_{\inf,B}\;T_{B,j}=P_{\inf,X}^{red}\;T_{X,j}^{red},\text{for}\;j=0\ldots N^{red}-1,\text{and}\;P_{\inf,A}+P_{\inf,B}=P_{\inf,X}^{red}\text{,}$$

giving

(10) $$T_{X,j}^{red}=\frac{P_{\inf,A}\;T_{A,j}+P_{\inf,B}\;T_{B,j}}{P_{\inf,A}+P_{\inf,B}}\text{,}$$

where *N*^*red*^ is the number of states in the reduced model, with transition matrix *T*^*red*^. Similarly, to preserve the ingoing probability flux, we impose

$$P_{\inf,i}\;T_{i,A}+P_{\inf,i}\;T_{i,B}=P_{\inf,i}\;T_{i,X}^{red},\text{for}\;i=0\ldots .N^{red}-1\text{,}$$

giving

(11) $$T_{i,X}^{red}=T_{i,A}+T_{i,B}\text{.}$$

Equations 10 and 11 can be applied multiple times to lump together as many states as desired. This reduction strategy is equivalent to starting from a count matrix containing all the transition events seen for the original *N* states model and summing up all the events starting from or ending at the lumped states into a single value, as well as summing all the transitions in and out of the lumped states. The same result would be obtained starting from MD data by redefining a single state including all the state spaces of the two lumped states. To assess the ion current using the reduced model, we also need to define a coherent reduced *F*, *F*^*red*^. As shown in section 4 of the supporting material, the knowledge of the mathematical relationship between the ion current and *T* and *F* matrices (Eq. 6) allows us to define *F*^*red*^ using the criterion of ingoing and outgoing flux charge conservation:

$$P_{\inf,A}\;T_{A,j}\;F_{A,j}+P_{\inf,B}\;T_{B,j}F_{B,j}=P_{\inf,X}\;T_{X,j}F_{X,j}^{red},\text{for}\;j=0\ldots N^{red}-1\text{,}$$

giving

(12a) $$F_{X,j}^{red}=\frac{P_{\inf,A}\;T_{A,j}\;F_{A,j}+P_{\inf,B}\;T_{B,j}F_{B,j}}{P_{\inf,A}\;T_{A,j}+P_{\inf,B}\;T_{B,j}}\text{,}$$

and

$$P_{\inf,i}\;T_{i,A}{\;F}_{i,A}+P_{\inf,i}\;T_{i,B}{\;F}_{i,B}=P_{\inf,i}\;T_{i,X}\;F_{i,X}^{red},\text{for}\;i=0\ldots N^{red}-1\text{,}$$

giving

(12b) $$F_{i,X}^{red}=\frac{T_{i,A}{\;F(∆t)}_{i,A}+T_{i,B}{\;F(∆t)}_{i,B}}{T_{i,A}+T_{i,B}}\text{.}$$

In the case of the term $F_{X,X}^{red}$, we need to consider possible charge fluxes existing between the lumped states:

$$P_{\inf,A}\;T_{A,A}\;F_{A,A}+P_{\inf,A}\;T_{A,B}\;F_{A,B}+P_{\inf,B}\;T_{B,A}F_{B,A}+P_{\inf,B}\;T_{B,B}F_{B,B}=P_{\inf,X}\;T_{X,X}F_{X,X}^{red}\text{,}$$

giving

(13) $$F_{X,X}^{red}=\frac{P_{\inf,A}\;T_{A,A}\;F_{A,A}+P_{\inf,A}\;T_{A,B}\;F_{A,B}+P_{\inf,B}\;T_{B,A}F_{B,A}+P_{\inf,B}\;T_{B,B}F_{B,B}}{\;P_{\inf,A}\;\left(T_{A,A}+T_{A,B}\right)+P_{\inf,B}\;\left(T_{B,A}+T_{B,B}\right)}\text{.}$$

Once the reduced *T*, *T*^*red*^, and the reduced *F*, *F*^*red*^, are obtained, using Eqs. 10, 11, 12a, 12b, and 13, the ion current of the reduced model can be predicted using an equation similar to Eq. 6:

(14) $$I≅\frac{e_{0}}{\Delta t}\sum_{i,j=0}^{N^{red}-1}P_{\inf,i}^{red}\;T_{ij}^{red}\left(\Delta t\right)F_{ij}^{red}\left(\Delta t\right)\text{.}$$

As an example, this reduction procedure was applied to lump together three states of the model in Fig. 1 *B* (KOKOOK, KOOKOK, and WKOKOK), as shown in Fig. 3 *B*. Fig. 3 *C* shows the current predicted for the full MSM (Eqs. 5a and 5b, *black line*) and for the reduced, four-state model (Eq. 14, *red line*). It is evident that the correct current is recovered, once again, provided that a sufficiently small lagtime is used for the determination of *T*.

##### The problem of non-Markovianity

**Figure 4:**
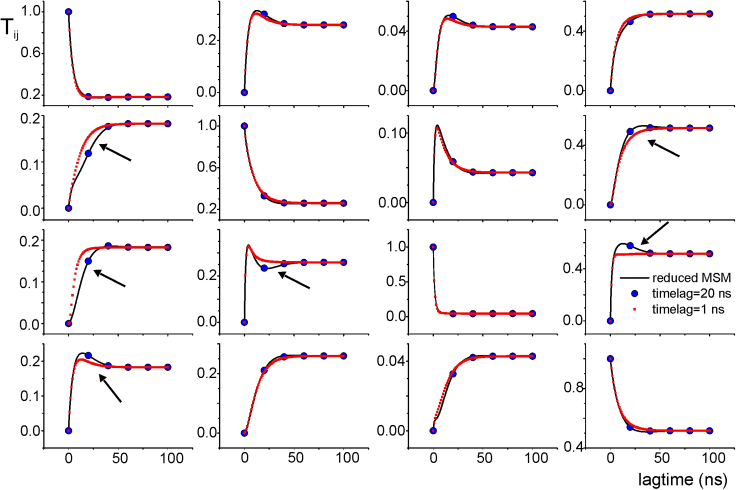
*T* elements obtained for the reduced MSM (the plot in *row i* and *column j* represents the *T*_*ij*_ element of the matrix) versus lagtime (*black lines*) are compared with the Chapman-Kolmogorov prediction using a lagtime of either 1 (*red symbols*) or 20 ns (*blue symbols*). Arrows indicate the largest discrepancies between the Chapman-Kolmogorov predictions and the values estimated from the simulated trajectories.

Previous sections demonstrate that the ion current is correctly predicted only when very short lagtimes are used for constructing the *T*s. Unfortunately, in the case of MD data, short lagtimes might lead to violation of the Chapman-Kolmogorov equation (4). The non-Markovianity in the low-lagtime regime may be explained by the presence of multiple energy minima (microstates) within the states chosen to build the MSM. If the transitions between the microstates are not sufficiently fast compared to the chosen lagtime, then the system can preserve memory of its previous (older than one lagtime) history, which makes it non-Markovian. The reduction procedure described above (Fig. 3) to decrease the number of states might aggravate the problem. In fact, in the new reduced model, macrostates lump together different microstates, and this limits the Markovianity at lagtimes sufficiently larger than the lifetime of the single merged microstates. In Fig. 4, the black continuous lines represent the *T* elements (the element *T*_*ij*_ is represented by the plot in *row i* and *column j*) obtained for the reduced model, using Eqs. 10 and 11 on full model *T* taken at various lagtimes. Red and blue symbols in the plots represent instead the prediction of the Chapman-Kolmogorov equation (Eq. 4) performed using *T*s of the reduced model obtained for lagtimes of 1 and 20 ns, respectively. It is evident that significant deviations from the Markovian prediction are present when the lagtime considered is 1 ns (arrows indicate the major discrepancies), while the predictions for a lagtime of 20 ns are essentially superimposed to the behavior of the reduced model. These results are in line with the notion that the lumping of different microstates generates a non-Markovian behavior of the resulting reduced model, especially evident at short lagtimes.

The non-Markovianity at short lagtimes represents a major limitation when trying to predict ion currents. As described in the previous paragraph, the prediction of an ion current can be done by first constructing an *F* valid only at very short lagtimes and then progressively/recursively assessing composite *F*s valid for larger lagtime *T*s, by performing a weighted mean of fluxes resulting from all the possible pathways. However, it is important to note that, when using Eqs. 7 and 8 to recursively assess the composite *F*, we are assuming a Markovian behavior of the system (Eq. 7 is a different form of the Chapman-Kolmogorov equation), and thus this procedure will not produce a correct result in the case of a reduced, non-Markovian model. This is demonstrated in Fig. 3 *C*, where the dashed blue line, obtained using Eqs. 7, 8, and 9 assuming Markovian behavior, slightly but significantly deviates from the correct value of the current. A possible solution to this problem is to consider the *T*s directly obtained at various lagtimes from MD data instead of predicting them by assuming a Markovian behavior and using Eq. 7. Along this line, we propose the use of the following equation to calculate the composite *F*:

(15) $$F_{ij}\left(n\Delta t\right)=\frac{\sum_{m=0}^{N-1}T_{im}^{*}\left(\left(n-1\right)\Delta t\right)*T_{mj}\left(\Delta t\right)*[F_{i,m}\left(\left(n-1\right)\Delta t\right)+F_{mj}\left(\Delta t\right)]}{T_{ij}^{*}\left(n\Delta t\right)}\text{,}$$

where *T*^∗^ represents *T*s calculated directly from simulated trajectories at varying lagtimes instead of using Eq. 7. The green dashed line in Fig. 3 *C* demonstrates that this procedure recovers the correct current at all lagtimes.

#### Assessing MSM rate constants of ion permeation

**Figure 5:**
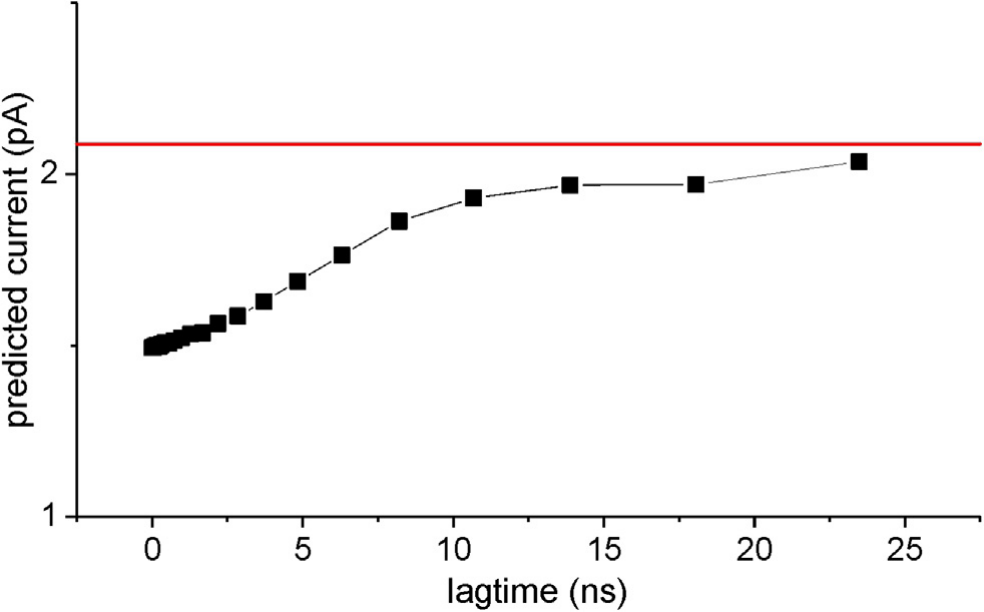
Currents estimated using Eqs. 5a and 5b, with a *Q* matrix recovered from the fitting procedure (Eq. 16) where reduced transition probability matrices at varying lagtimes were used. It is evident that a correct current is predicted only when transition probability matrices obtained at large lagtimes are used in the minimization procedure. The red line represents the correct value of the current for the model considered.

The methods described above allow us, starting from simulated trajectories, to obtain an MSM for ion permeation containing relatively few states, with a *T* and associated *F* at lagtimes at which the model is fully Markovian. In case that the *Q* matrix is also needed, this can be estimated using a fitting procedure that minimizes the following function:

(16) $$f=\left|T^{red}\left(\Delta t\right)-\mathrm{Exp}\;\left(Q^{red}\Delta t\right)\right|\text{,}$$

where $T^{red}\left(\Delta t\right)$ is the *T* of the reduced model, obtained as described at the lagtime Δt, and $Q^{red}$ contains the rate constants to be identified. In our reduced model, the estimated *Q* matrix returned, using Eq. 5b, a current of 1.98 pA, close to the real value of 2.09 pA. Notice that the minimization of Eq. 16, based on the relation between *T* and *Q* matrices given in Eq. 2, recovers the correct *Q* matrix only for a fully Markovian MSM. This implies that the minimization needs to be performed using *T*s obtained at lagtimes sufficiently large to guarantee a Markovian behavior. This is shown in Fig. 5, which reports the current predicted using a *Q* matrix obtained from *T*s at varying lagtimes. It is evident that the prediction based on the *Q* matrix improves when increasing the lagtime and, consequently, when the Markovian behavior is more and more satisfied (cf. Fig. 4 *C*).

Given the relatively large number of free parameters in the minimization procedure, we noted that the assessment of the *Q* matrix could find problems related to multiple minima in the function. We found that a regularization can be obtained by adding to the function to be minimized a term proportional to the difference between the current predicted by *T* and the current predicted by the estimated *Q*, found with Eqs. 5b and 6, respectively,

(17) $$f=\left|T^{red}\left(\Delta t\right)-\mathrm{Exp}\;\left(Q^{red}\Delta t\right)\right|+\lambda *|I_{T}-I_{Q}|\text{,}$$

where $I_{T}$ and $I_{Q}$ represent the current predicted from the *T* and the *Q* matrix, respectively, and *λ* is a weighting factor.

#### Suggested algorithm

To summarize, we propose the following procedure to extract a reduced MSM from MD data.

(1) Estimate *T*s for ion permeation at varying lagtimes from MD data.
(2) Define the *F* associated with the shortest lagtime *T* available using the algorithm in the supporting material. The use of a sufficiently short lagtime can be verified by comparing the current predicted by the transition and flux matrices (cf. Eq. 6) with the ion current directly assessed by MD simulations. A predicted current significantly different from the one obtained from MD is indicative of a too-large lagtime.
(3) Reduce the model at short lagtimes by lumping together states and finding reduced *T*s and flux matrices through Eqs. 10, 11, 12a, 12b, and 13. The correctness of the reduction procedure can be verified by monitoring the current predicted by the reduced model at the short lagtime using Eq. 14. Different strategies of model reduction can be considered, based on the selection of the most physically significant states, the relevant state occupancy, spectral clustering, and other parameters.
(4) Test the reduced model for Markovianity of the *T*. In case of failure, increase the lagtime until a nearly Markovian behavior is found. Assess flux matrices at large lagtimes, at which the model is fully Markovian, using Eq. 15. In this way, it will be possible to use the model for predicting the ion current from the *T* obtained at large lagtimes.
(5) Assess the rate constants associated with each transition of the reduced model using a fitting procedure that maximizes Eq. 16 or 17 and a fully Markovian *T*, obtained at large lagtimes.

The correctness of the procedure can be monitored by comparing at the various stages the predicted ion current with the one directly computed by counting ion crossing events in simulated trajectories.

## Results

We used the above algorithm to find a reduced model of K permeation through the KcsA channel from *T*s built at various lagtimes using MD data at +200 mV of membrane potential. We performed a total of 29.69 *μ*s of simulation time in eight replicas, obtaining 334 permeation events, resulting in a current of 1.79 pA. As reported by Domene et al. (8), the *T* was determined by looking at the presence of K and/or water within the six different sites S5 to S0 (going from the intracellular cavity to the extracellular side of the SF). Of the whole set of possible ion and water configurations, only 122 were present in the original *T*, i.e., had been effectively sampled in the MD simulations.

**Figure 6:**
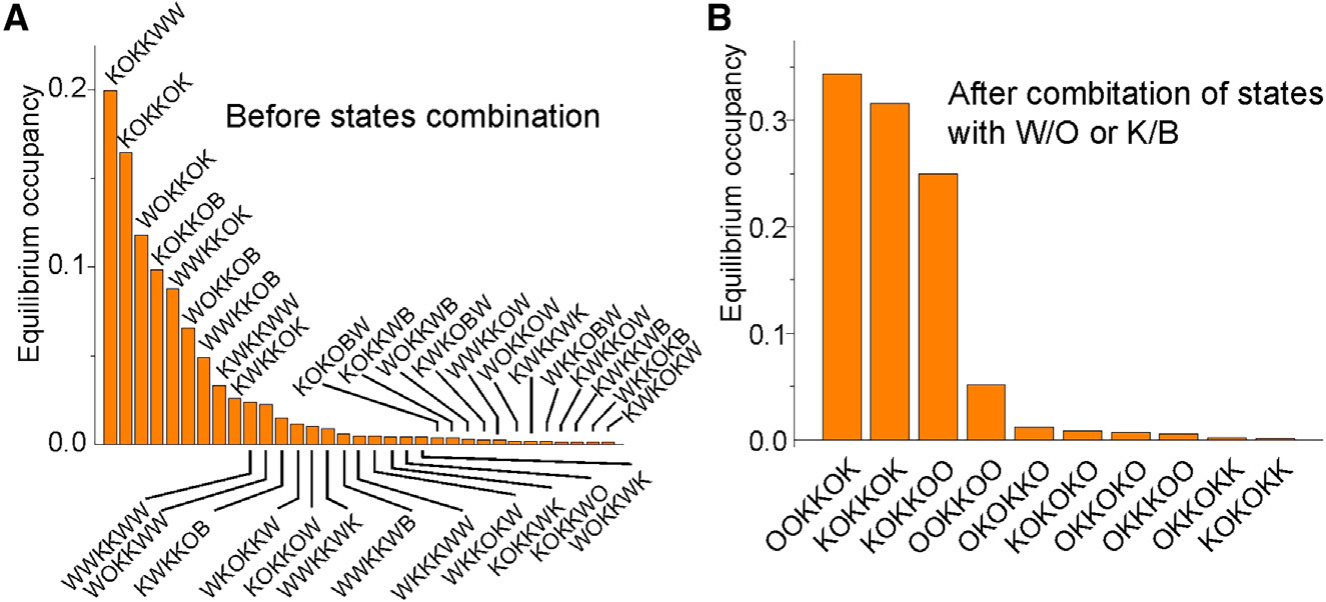
(*A* and *B*) Plots of the equilibrium occupancy of the various state configurations found in the MD data before (*A*) and after (*B*) lumping together states that have the same K ions configurations, regardless of the presence of water in the binding sites. In both cases, only states with occupancy higher than 0.1% are shown.

Fig. 6*A* shows the occupancy of the various filter configurations at steady state (only the configurations with an equilibrium occupancy higher than 0.1% are shown). The result is that the most stable configurations are those having a K ion at sites S0, S2, S3, and S5, with sites S2 and S3 always occupied, regardless of the presence of water in the various sites.

Since we were mainly interested in the permeation of K, we performed a combination of states that had the same K ion configuration. As an example, states KOKKOK, KOKKOB, KOKKWK, KOKKWB, etc., were all combined in one configuration indicated as KOKKOK, where, in this case, K indicates the presence and O the absence of a K ion in that site, regardless of the presence of water. As reported above, state combination was performed using a method that preserves the steady-state occupancy of the remaining states, as well as ingoing and outgoing ion fluxes. The described combination allowed us to reduce the number of states from 122 to 27, as shown in decreasing order of steady-state occupancy in Fig. 6 *B* (note that only states with occupancy higher than 0.1% are shown). Once again, it is possible to notice that the most represented states are those having sites S0, S2, S3, and S5 occupied by K, while only rarely do sites S1 and S4 bind a permeating ion.

**Figure 7:**
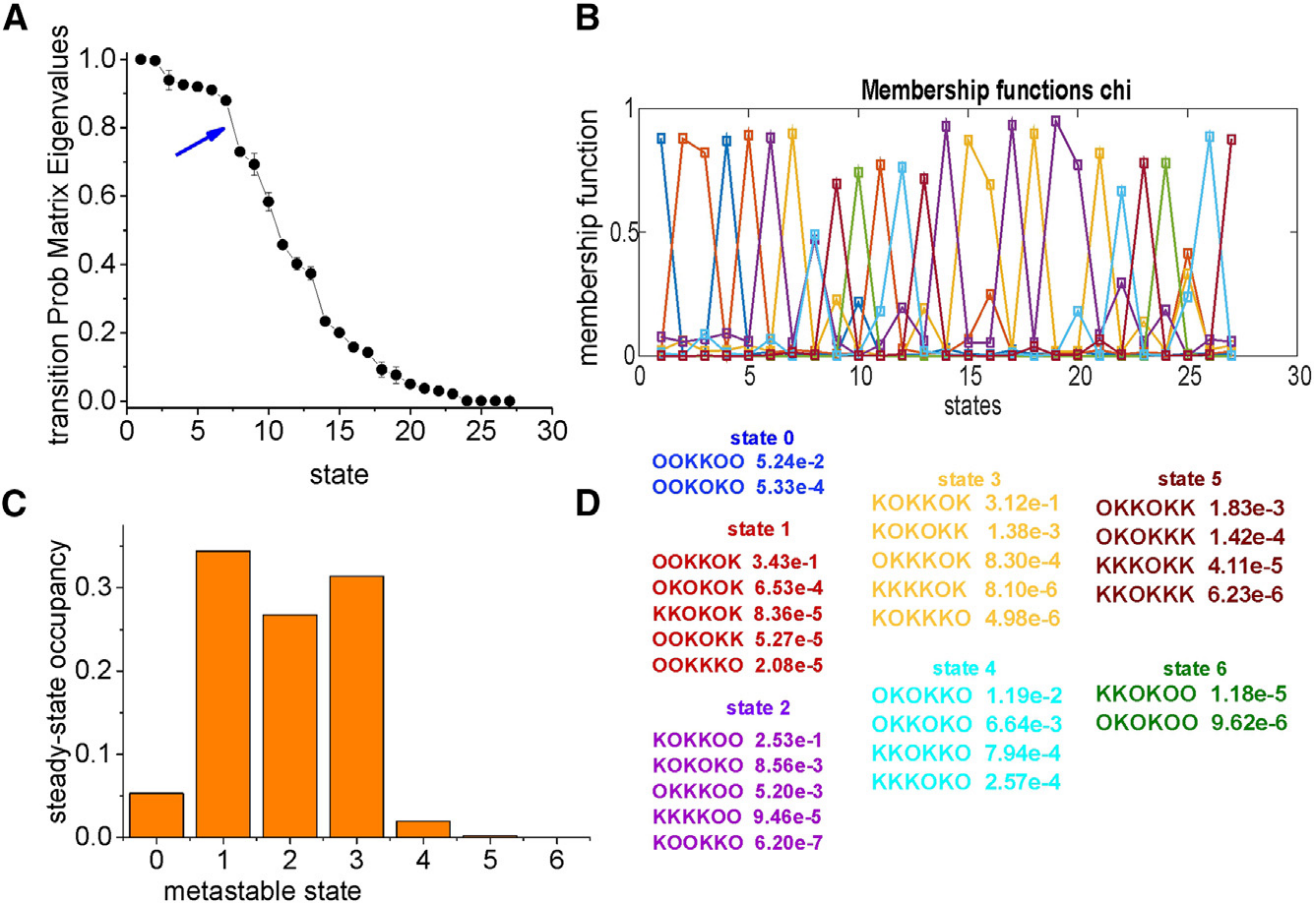
(*A*) Module of the eigenvalues of the *T* associated with the partially reduced Markov model (cf. Fig. 6*B*); the blue arrow indicates a gap separating the first seven eigenvalues from the following ones. (*B*) Membership function returned by the cPCCA+ algorithm. Different colors are used for the membership function of the seven macrostates, whose state compositions and steady-state occupancy are reported in (*D*). (*C*) Equilibrium occupancy of the seven macrostates identified using the cPCCA+ algorithm.

Out of the 27 resulting states, we finally performed a further combination of ion configurations using a *T* spectral clustering based on the Perron cluster algorithm (PCCA+) (30). Since ion conduction is an irreversible process, we used a variant of the PCCA+ algorithm, the cPCCA+, which can handle complex eigenvalues, typical of irreversible *T*s (31). Fig. 7 *A* shows the module of the eigenvalues of the *T* obtained after combining states with the same K ion configuration. An evident gap can be identified between the seventh and eighth eigenvalues (arrow), suggesting that the clustering of the 27 states into seven macrostates produces sufficiently well-separated kinetics between micro- and macrostates. Consequenlty, the model was clustered into seven macrostates (denominated states 0–6) using the cPCCA+ algorithm. Fig. 7 *B* shows the fuzzy clustering of the 27 states into seven clusters according to the membership functions, with the different colors indicating the membership function for the different macrostates, and Fig. 7 *D* shows the microstates (configurations) included in the seven different macrostates.

**Figure 8:**
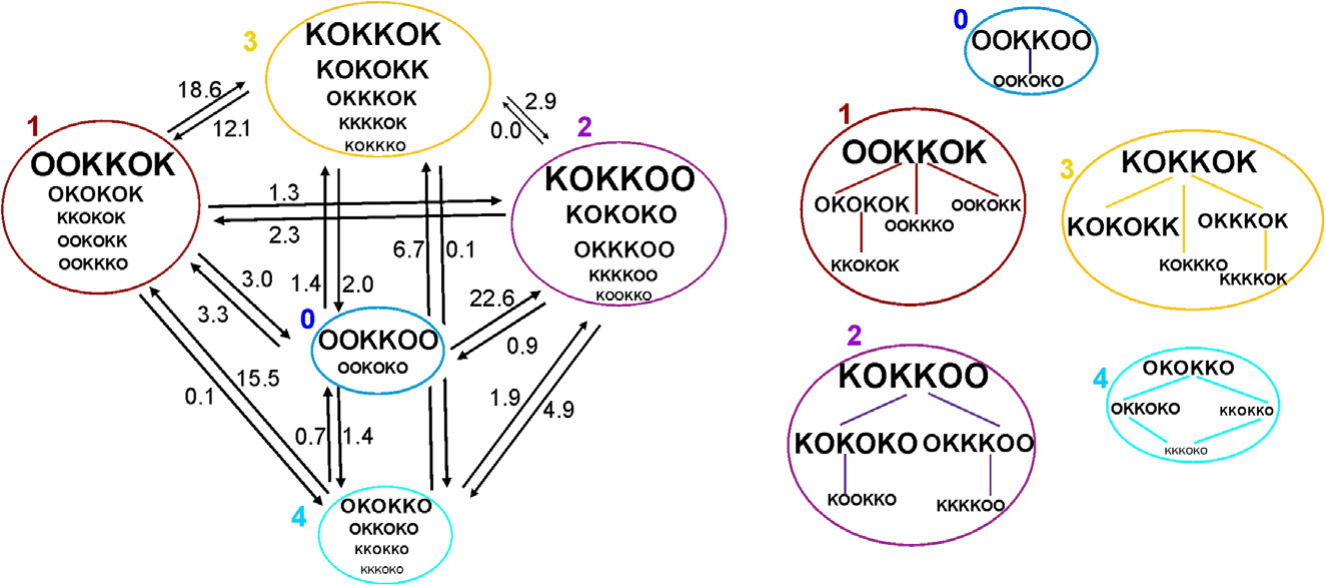
(*Left*) Kinetic scheme representing the reduced model, with the 5 macrostates marked with the same color code as in Fig. 7. The numbers next to each arrow represent the kinetic rate constants associated with the various transitions in units of 10^7^ s^−1^. Within each macrostate, the different microstates are shown with a size approximately corresponding to its steady-state occupancy. (*Right*) Microstates belonging to the same macrostate and differing for the movement of a K ion from a binding site to an adjacent one are connected with a line.

Finally, we performed a fit of the *T* obtained for a lagtime of 10 ns to obtain the transition rate constants and the connectivity of the MSM of K permeation. The numbers in the scheme of Fig. 8 report the best-fit parameters obtained. As shown in Fig. 7 *C*, two of the seven macrostates, states five and six, exhibit a very low steady-state occupancy, and consequently, for the sake of clarity, they were not included in Fig. 8, which shows the kinetic scheme of the reduced model, together with the microstates contributing to each macrostate of the model. To note, the resulting model is very similar to the association-dissociation model previously proposed (14,32). In particular, permeation appears to follow two distinct routes. A first one generates conductance by the association of a K ion to the internal binding site, a sort of single-file movement of the three K ions so as to expose a bound K ion to the external site and induce its dissociation. The second route of permeation involves the binding of a K ion to the internal site, the subsequent knock on of the other ions toward the external side, and the exit of the most external K.

**Figure 9:**
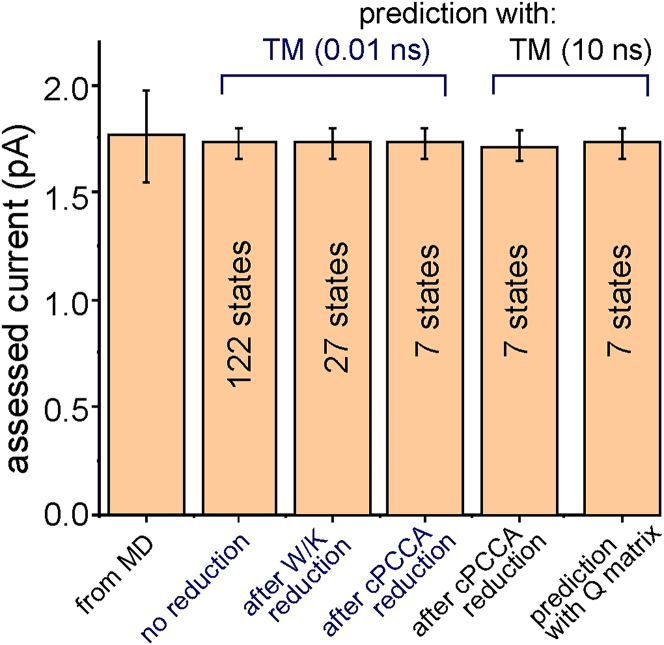
Comparison of the current assessed directly from MD (mean ± standard deviation for eight replicas) with currents predicted by the full and reduced MSMs, with both the *T* matrix and the *Q* matrix.

Once a reduced MSM model of K permeation through the KcsA channel was obtained, we estimated the *T* and the corresponding *F* at higher lagtimes, using the method described in the previous section, to have a fully Markovian model of K permeation. The system exhibits an almost Markovian behavior for lagtimes above 5–10 ns (see section 5 of the supporting material), and consequently, a lagtime of 10 ns was used. As shown in Fig. 9, our method of assessment of the composite *F* at higher lagtimes predicted a current like that directly obtained from MD, suggesting that the method used was robust. As a further test of the entire procedure, the *F* elements of the reduced model, as estimated using the method outlined in the previous section, were compared to the corresponding values directly calculated from the MD trajectories (section 6 of the supporting material). The two procedures returned similar values for the *F* elements, confirming the validity of the method.

## Discussion

The method reported in this paper integrates MD simulations and MSMs to advance the understanding of ion channel permeation. Specifically, our approach enables the construction of a reduced MSM for ion channel permeation, characterized by a relatively low number of states, while still being capable of quantitatively predicting ion currents at any lagtime in a very accurate way. This model serves two primary purposes: comparing MD results with experimental data and elucidating the ion channel permeation mechanism by identifying the key ion movements within the channel pore.

An MSM approach able to derive the ion current passing through the channel has been previously reported by de Groot and colleagues (9). In that study, the current through the channel was obtained from the total number of net ion jumps in the SF throughout a simulation. Here, we instead developed a mathematical relationship that directly relates the *T* and the *F* of the MSM to the ion current. The availability of a mathematical relationship relating the ion current to the properties of the MSM is a necessary step for enabling further analyses of MSM-based conduction mechanisms. For example, this has allowed us to validate a procedure that groups different states of the original MSM while retaining the same ion current and thus define an MSM with fewer metastable states. This condition greatly facilitates the mechanistic understanding of permeation while accurately predicting the ion current. This method can also facilitate quantitative comparisons between different channels and boundary conditions. Mapping the dynamics onto a limited number of states is a hallmark of the literature using MSMs to analyze MD trajectories. In many cases, this mapping—and the intuitive understanding of the system that follows—is the primary reason MSMs are employed. However, the mapping procedure is meaningful only if the reduced MSM can still be used to predict relevant experimental properties, such as channel current. The methods presented here achieve exactly this: to calculate the ion current from MSMs regardless of the lagtime or the strategy adopted to reduce the number of states.

We applied the method to MD simulation data obtained at 200 mV, in the presence of 200 mM K, for one of the most studied K channels, KcsA, and found that it is not only able to reproduce the amplitude of current as directly estimated from MD trajectories but can also suggest a very specific K permeation mechanism. Following the state reduction procedure described, we obtained an MSM with only five main states, and *T*s and *F*s capable of predicting essentially the same ion current directly measured in the MD simulation, by counting the number of K ions passing through the pore. It is important to remark that the method is based on the assumption that the initial *T* and *F*, i.e., the ones estimated at the shortest lagtime, correctly represent the transitions among the different configurations without missing events due to multiple transitions. In the opposite case, this initial lagtime needs to be reduced. Once the correct initial lagtime is defined, the proposed method can be used to estimate the correct *F* for reproducing the current at any lagtime. The results of the analysis suggest that ion permeation in KcsA follows an association-dissociation scheme, meaning that the binding/unbinding of a K ion at one entrance of the channel promotes the unbinding/binding of a K ion at the other entrance. The two processes, combined with the ability of K ions inside the SF to move in single file, generate a cyclic process promoting the continuous passage of permeant ions from one side of the membrane to the other.

The present KcsA model has been built using MD data with only one ion (symmetrical 200 mM KCl) and voltage (200 mV) condition. The construction of an MSM valid under other conditions and able to predict current-voltage (IV) relationships will need additional MD data. The reduced model presented here could be useful to assess how the ion current depends on the value of each kinetic rate constants and how these rate constants depend on boundary conditions. In fact, since the five macrostates of the model represent clusters of different ion configurations, the dependence of each rate constant on the intracellular and/or extracellular ion concentration and membrane potential may be complex and not linear, as it was usually assumed in simple permeation kinetic schemes used in the pre-structural times. Future MD studies are needed to investigate these relationships and expand the predictive power of MSMs in ion channel applications.

A major advantage of the proposed method is to link the atomic structure of the ion channel pore to the main experimental data in ion channel permeation, i.e., the single-channel current. Although ion currents can be estimated using MD simulation, the high computational cost involved limits the range of analyses and imposes the use of extreme boundary conditions in terms of membrane potentials and ion concentrations. Based on the methods proposed here, it is possible to envision a strategy where MD simulations at a limited number of boundary conditions are used to estimate reduced MSMs, which are then used to predict how the kinetic rate constants depend on the boundary conductions and, consequently, the ion currents at conditions that were not included in the original set of MD simulations. The application of our proposed method would, in addition, greatly facilitate the understanding of the main determinants of the permeation mechanisms in a variety of ion channels.

Currently, MD produces such an overwhelming amount of data on permeation processes that it is extremely difficult to identify their main determinants. The approach of analyzing MD data to obtain MSMs of ion channel permeation, and then repeatedly applying the state reduction technique proposed in this paper, would allow the identification of the few relevant metastable states and the few transitions that primarily define the permeation process. Many relatively simple kinetic schemes, which are essentially reduced Markov models, were proposed in the second half of the last century to explain the permeation and selectivity of many different ion channels (33,34,35,36). These simple mechanisms have been extensively tested for their ability to accurately reproduce many experimental results. The approach proposed in this paper could be used, once the atomic structures of those channels have been determined, to understand, in terms of single amino-acid residues and atomic structure, the significance of the states and transitions present in each of these kinetic models.

## Acknowledgments

Funded by the European Union - NextGenerationEU under the National Recovery and Resilience Plan (PNRR) - Mission 4 Education and Research - Component 2 From Research to Business-Investment 1.1, Notice Prin 2022 - (DD N. 104 del 2/2/2022) title “Kinetic models of ion channels: from atomic structures to membrane currents”, proposal code 20223XZ5ER - CUP J53D23006940006. S.F. acknowledges CINECA for awarding access to computational resources through the ISCRA Initiative (grant number HP10BJPCFW).

## Author contributions

S.F. and L.C. designed the research; A.M., M.V.L., C.D., and F.F. contributed to the design of the research and interpretation of data; C.D. and S.F. performed MD; L.C. and S.F. wrote the initial draft of the paper; and all authors contributed to and approved the final form of the paper.

## Declaration of interests

The authors declare no competing interests.

## Supporting material

Document S1. Figures S1–S5 and supporting materials and methods

Document S2. Article plus supporting material
