# Supplementary material for: Building predictive Markov models of ion channel permeation from molecular dynamics simulations
Source: Biophys J. 2024 Sep 28;123(21):3832–43. doi: 10.1016/j.bpj.2024.09.030 (PMC11630636; doi:10.1016/j.bpj.2024.09.030)
Supplement: Document S1. Figures S1–S5 and supporting materials and methods [file mmc1.pdf]

Biophysical Journal, Volume *123*

Supplemental information

Building predictive Markov models of ion channel permeation from molecular dynamics simulations

Luigi Catacuzzeno, Maria Vittoria Leonardi, Fabio Franciolini, Carmen Domene, Antonio Michelucci, and Simone Furini

Supplementary Material

Building predictive Markov State Models of ion channel permeation from Molecular Dynamics Simulations

Luigi Catacuzzeno[1], Maria Vittoria Leonardi[1], Fabio Franciolini[1], Carmen Domene[2], Antonio Michelucci[1], Simone Furini[3]

[1]Department of Chemistry, Biology and Biotechnology, University of Perugia, Italy

[2] Department of Chemistry, University of Bath, Claverton Down, Bath, BA2 7AY, UK

[3] Department of Electrical, Electronic and Information Engineering "Guglielmo Marconi", University of Bologna, via dell'Università 50, Cesena (FC), 47521, Italy

1. Relationship between the transition probability matrix, T, and the Q matrix for a continuous time Markov process

A stochastic process Z(t), defined over a discrete state space S of cardinality N, is a continuous time Markov process (CTMP) if for any sequence of times (t_0<t_1.......<t_{m-1}<t_m):

$$Prob\left\{Z(t_m) = s_j \middle| Z(t_0) = s_0, Z(t_1) = s_1 \dots\dots\dots\dots.. Z(t_{m-1}) = s_{m-1}\right\}$$
$$= Prob\left\{Z(t_m) = s_j \middle| Z(t_{m-1}) = s_{m-1}\right\}$$

Where $Prob\left\{Z(t_m) = s_j \middle| Z(t_{m-1}) = s_{m-1}\right\} = T_{ij}(t_{m-1}, t_m)$ are the elements of the matrix of conditional probabilities T(t_{m-1},t_m), and $Prob\left\{Z(t) = s_j\right\} = p_i(t)$ is the row vector of occupancies p(t).

The Chapman-Kolmogorov (C-K) equations implies that:

$$p(t_2) = p(t_1)\, P(t_1, t_2)$$

$$T(t_1, t_3) = T(t_1, t_2) T(t_2, t_3)$$

A CTMP is time homogeneous if the conditional probability T(t_1,t_3) depends only on the difference t_3-t_1, i.e. T(t_1,t_3)= T(t_1+ t_2,t_3+ t_2)=T(t_3- t_1). In this case the C-K equation becomes:

$$p(t + \Delta t) = p(t)\, T(\Delta t)$$

$$T(t + \Delta t) = T(t) T(\Delta t)$$

If we define the Q matrix with elements $q_{ij} = \frac{T_{ij}}{\Delta t}$ for small Δt, and $q_{ii} = -\sum_j q_{ij}$, it follows that $T_{ij}(\Delta t) = q_{ij}\, \Delta t$ and $T_{ii}(\Delta t) = 1 + q_{ii}\Delta t$.

The homogeneous C-K equation then becomes:

$$T_{ij}(t + \Delta t) = \sum_k T_{ik}(t)\, T_{kj}(\Delta t) = T_{ij}(t)\, T_{jj}(\Delta t) + \sum_{k \neq j} T_{ik}(t)\, T_{kj}(\Delta t)$$
$$= T_{ij}(t)\, (1 + q_{jj}\Delta t) + \sum_{k \neq j} T_{ik}(t)\, q_{kj}\, \Delta t$$

It follows:

$$\frac{T_{ij}(t + \Delta t) - T_{ij}(t)}{\Delta t} = T_{ij}(t)\, q_{jj} + \sum_{k \neq j} T_{ik}(t)\, q_{kj} = \sum_k T_{ik}(t)\, q_{kj}$$

$$\frac{dT(t)}{dt} = P(t) Q$$

The above equation has the formal solution:

$$T(t) = e^{Q\, t}$$

relating the transition probability matrix to the Q matrix.

2. Algorithm to define the flux matrix at the shortest lagtime

1) Starting from an initial configuration i, other configurations are produced by allowing the entrance of water or K ions to the external sites S0 and S5, or moving water and K ions from a binding site to the adjacent binding sites inside the SF in both directions.

2) For each new configuration, an associated flux is assessed by adding +1/7 for a K+ movement towards the external side, and -1/7 for a K+ movement towards the internal side. For example, a flux of 1/7 is assigned to the transition KOKKOO → KOKOKO since the configuration KOKOKO is obtained by moving the K ion from S2 to S1.

3) Point 2 is repeated until configuration j is reached, and the i → j flux is assessed by summing all the fluxes associated to the intermediate transitions while going from i to j. For example, a flux of +2/7 is associated to the transition KOKKOO → KOKOOK if this transition is obtained by two consecutive transitions KOKKOO → KOKOKO and KOKOKO → KOKOOK each having a flux of +1/7.

3. Derivation of the equation used to estimate the flux matrix, F, at increasing lagtimes

The element $F_{ij}(n\Delta t)$ of the flux matrix corresponds to the fractional number of conduction events in the outward direction that occurs when the system moves from state i to state j in a time interval nΔt. The time interval nΔt can be divided into two successive time intervals: (n-1)Δt and Δt. Using these discrete temporal steps, the transition from state i to state j can be described as two successive events: first from state i to state m, and then from state m to state j, with m being any one of the possible N states of the system (as schematically depicted in Figure S1). Therefore, $F_{ij}(n\Delta t)$ can be estimated as:

$$F_{ij}(n\Delta t) = \sum_{m=0}^{N-1} p_{ij}^{m}(n\Delta t) F_{ij}^{m}(n\Delta t)$$

Where $p_{ij}^{m}(\Delta t)$ is the probability that a system moving from state i to state j in the time interval nΔt is in state m at time (n-1)Δt; and $F_{ij}^{m}(n\Delta t)$ is the fractional number of conduction events in the outward direction that occurs when the system moves from state i to state j in a time interval nΔt following a route that passes through state m at time (n-1)Δt. The number of conduction events along this route passing through m can be written as:

$$F_{ij}^{m}(n\Delta t) = F_{im}((n-1)\Delta t) + F_{mj}(\Delta t)$$

Here, $F_{im}((n-1)\Delta t)$ is the number of conduction events for the transition i → m in $(n-1)\Delta t$ and $F_{mj}(\Delta t)$ is the number of conduction events for the transition m → j in Δt. Since Markovian behavior is assumed, the transitions i → m and m → j are uncorrelated, and $p_{ij}^{m}(\Delta t)$ is equal to:

$$p_{ij}^{m}(n\Delta t) = \frac{T_{im}((n-1)\Delta t) T_{mj}(\Delta t)}{T_{ij}(n\Delta t)}$$

Using the last two equations in the previous one gives:

$$F_{ij}(n\Delta t) = \frac{\sum_{m=0}^{N-1} T_{im}((n-1)\Delta t) T_{mj}(\Delta t)[F_{im}((n-1)\Delta t) + F_{mj}(\Delta t)]}{T_{ij}(n\Delta t)}$$

Which corresponds to equations 8 and 15 of the main manuscript. The difference between equation 8 and 15 is that in the first case $T_{ij}(n\Delta t)$ is estimated using the Chapman-Kolmogorov equation starting from $T(\Delta t)$, while in equation 15, it is directly estimated from simulated trajectories. In both cases, the flux matrices at increasing lagtimes are calculated with a step-by-step procedure. Firstly, F(2Δt) is calculated from F(Δt), T(Δt), and T(2Δt). Then, F(3Δt) is calculated from F(2Δt), F(Δt) , T(2Δt), T(Δt), and T(3Δt), and likewise for the following lagtimes.

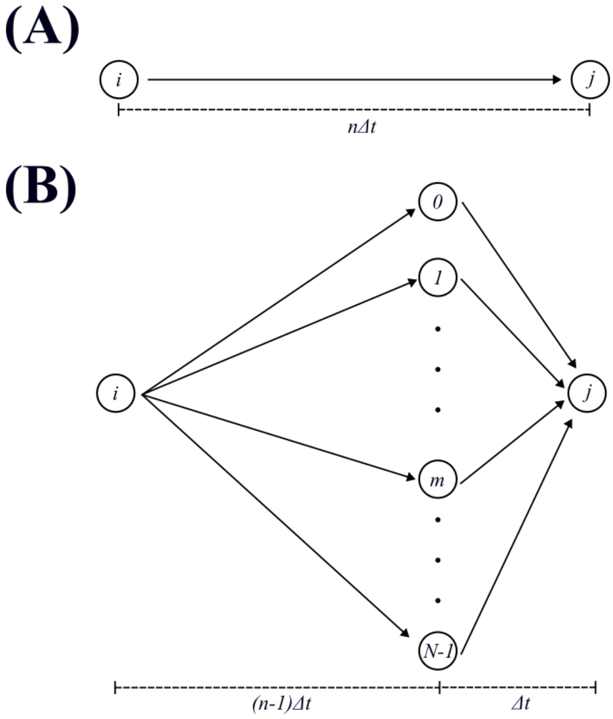

Figure S1. Schematic representation of the procedure used to calculate the flux matrix at time nΔt. The transition from i to j in the time interval nΔt **(A)** is separated into N possible alternative routes, according to the state of the system at time (n-1) Δt **(B)**.

4. Derivation of the flux matrix for the reduced MSM

We consider the process of lumping two states of the MSM, A and B, into a single state X (cf Figure 3A of the main text). To preserve the outgoing and ingoing probability fluxes of the reduced MSM, the X to j and j to X elements of the reduced transition matrix T^{red} need to be set to:

$T^{red}_{X,j} = \frac{p_{inf,A}\ T_{A,j} + p_{inf,B}\ T_{B,j}}{p_{inf,A} + p_{inf,B}}$ and $T^{red}_{i,X} = T_{i,A} + T_{i,B}$, with j going from 0 to N^{red}-1.

With this choice for the reduced transition matrix, we can assess the flux matrix that preserves the ionic current predicted by the model using the expression relating the ion current to T and F (eqn. 6).

More specifically, we impose that the current predicted by the original MSM is equal to the current predicted by the reduced model:

$$\frac{e_0}{\Delta t}\sum_{i,j=0}^{N-1} P_{inf,i}\ T_{ij}(\Delta t)F_{ij}(\Delta t) = \frac{e_0}{\Delta t}\sum_{i,j=0}^{N^{red}-1} P_{inf,i}^{red}\ T_{ij}^{red}(\Delta t)\ \ F_{ij}^{red}(\Delta t)$$

Considering that all the elements $T_{ij}(\Delta t)$ and $F_{ij}(\Delta t)$ with i ≠ A, B and j ≠ A, B are identical to the corresponding elements of the reduced matrix $T_{ij}^{red}(\Delta t)$ and $F_{ij}^{red}(\Delta t)$, we obtain the following relationship:

$$\sum_{\substack{i=0\\ i\neq A,B}}^{N-1} P_{inf,i}\ [T_{iA}(\Delta t)F_{iA}(\Delta t) + T_{iB}(\Delta t)F_{iB}(\Delta t)] + \sum_{\substack{j=0\\ j\neq A,B}}^{N-1} \left[P_{inf,A}\ T_{Aj}(\Delta t)F_{Aj}(\Delta t) + P_{inf,B}\ T_{Bj}(\Delta t)F_{Bj}(\Delta t)\right] + P_{inf,A}\ T_{AA}(\Delta t)F_{AA}(\Delta t) + P_{inf,B}\ T_{BB}(\Delta t)F_{BB}(\Delta t) + P_{inf,A}\ T_{AB}(\Delta t)F_{AB}(\Delta t) + P_{inf,B}\ T_{BA}(\Delta t)F_{BA}(\Delta t) = \sum_{\substack{i=0\\ i\neq X}}^{N^{red}-1}\left[P_{inf,i}^{red}\ T_{iX}^{red}(\Delta t)\ \ F_{iX}^{red}(\Delta t)\right] + \sum_{\substack{j=0\\ j\neq X}}^{N^{red}-1}\left[P_{inf,X}^{red}\ T_{Xj}^{red}(\Delta t)\ \ F_{Xj}^{red}(\Delta t)\right] + P_{inf,X}^{red}\ T_{XX}^{red}(\Delta t)\ \ F_{XX}^{red}(\Delta t)$$

Recalling that $T_{X,j}^{red} = \frac{P_{inf,A}\ T_{A,j} + P_{inf,B}\ T_{B,j}}{P_{inf,A} + P_{inf,B}}$ and $T_{i,X}^{red} = T_{i,A} + T_{i,B}$, and $P_{inf,X}^{red} = P_{inf,A} + P_{inf,B}$, we obtain:

$$\sum_{\substack{i=0\\ i\neq A,B}}^{N-1} P_{inf,i}\ [T_{iA}(\Delta t)F_{iA}(\Delta t) + T_{iB}(\Delta t)F_{iB}(\Delta t)] + \sum_{\substack{j=0\\ j\neq A,B}}^{N-1} \left[P_{inf,A}\ T_{Aj}(\Delta t)F_{Aj}(\Delta t) + P_{inf,B}\ T_{Bj}(\Delta t)F_{Bj}(\Delta t)\right] + P_{inf,A}\ T_{AA}(\Delta t)F_{AA}(\Delta t) + P_{inf,B}\ T_{BB}(\Delta t)F_{BB}(\Delta t) + P_{inf,A}\ T_{AB}(\Delta t)F_{AB}(\Delta t) + P_{inf,B}\ T_{BA}(\Delta t)F_{BA}(\Delta t) = \sum_{\substack{i=0\\ i\neq X}}^{N^{red}-1}\left[P_{inf,i}^{red}\ T_{iX}^{red}(\Delta t)\ \ F_{iX}^{red}(\Delta t)\right] + \sum_{\substack{j=0\\ j\neq X}}^{N^{red}-1}\left[P_{inf,X}^{red}\ T_{Xj}^{red}(\Delta t)\ \ F_{Xj}^{red}(\Delta t)\right] + P_{inf,X}^{red}\ T_{XX}^{red}(\Delta t)\ \ F_{XX}^{red}(\Delta t) = \sum_{\substack{i=0\\ i\neq X}}^{N^{red}-1}\left[P_{inf,i}^{red}\left(T_{i,A}(\Delta t) + T_{i,B}(\Delta t)\right)\ \ F_{iX}^{red}(\Delta t)\right] + \sum_{j=0}^{N^{red}-1}\left[\left(P_{inf,A}\ \ T_{A,j}(\Delta t) + \ P_{inf,B}\ \ T_{B,j}(\Delta t)\right)\ \ F_{Xj}^{red}(\Delta t)\right] + \left[P_{inf,A}\ \left(T_{A,A}(\Delta t) + T_{A,B}(\Delta t)\right) + P_{inf,B}\ \left(T_{B,A}(\Delta t) + T_{B,B}(\Delta t)\right)\right]\ F_{XX}^{red}(\Delta t)$$

The above equation may obviously be satisfied by different forms of the reduced flux matrix. Among them, we chose the one satisfying the following three charge conservation conditions:

$$P_{inf,i}\ [T_{iA}(\Delta t)F_{iA}(\Delta t) + T_{iB}(\Delta t)F_{iB}(\Delta t)] = \left[P_{inf,i}^{red}\left(T_{i,A}(\Delta t) + T_{i,B}(\Delta t)\right)\ \ F_{iX}^{red}(\Delta t)\right], \forall\ i \neq A, B$$

$$P_{inf,A}\ \ T_{Aj}(\Delta t)F_{Aj}(\Delta t) + P_{inf,B}\ T_{Bj}(\Delta t)F_{Bj}(\Delta t) = \left(P_{inf,A}\ \ T_{A,j}(\Delta t) + \ P_{inf,B}\ \ T_{B,j}(\Delta t)\right)\ \ F_{Xj}^{red}(\Delta t)\ , \forall\ j \neq A, B$$

$$P_{inf,A}\ \ T_{AA}(\Delta t)F_{AA}(\Delta t) + P_{inf,B}\ T_{BB}(\Delta t)F_{BB}(\Delta t) + P_{inf,A}\ \ T_{AB}(\Delta t)F_{AB}(\Delta t) + P_{inf,B}\ T_{BA}(\Delta t)F_{BA}(\Delta t) = \left[P_{inf,A}\ \left(T_{A,A}(\Delta t) + T_{A,B}(\Delta t)\right) + P_{inf,B}\ \left(T_{B,A}(\Delta t) + T_{B,B}(\Delta t)\right)\right]\ F_{XX}^{red}(\Delta t)$$

A rearrangement of the last three equations gives the equations 12a, 12b, and 13 of the main text of the paper for the reduced flux matrix.

5. Identification of a lagtime for markovian behavior

In Figure S2, the black continuous lines represent the T elements (the element T_{ij} is represented by the plot in row *i* and column *j*) obtained for the reduced model as a function of the lagtime directly from the MD trajectories. Black and red symbols in the plots represent instead the prediction of the Chapman-Kolmogorov equation performed using Ts of the reduced model obtained for lagtimes of 1 and 10 ns, respectively.

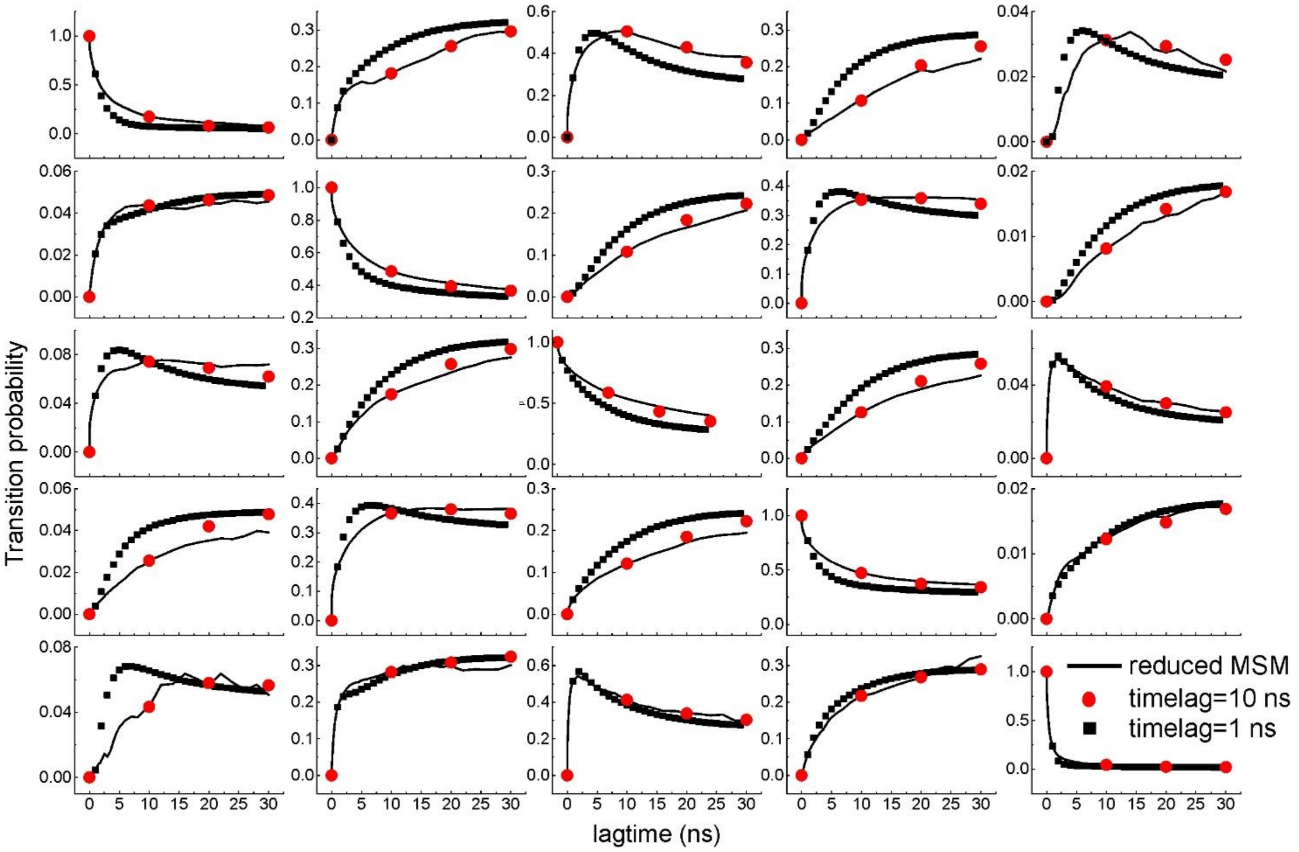

Figure S2. Transition matrix obtained for the MD data at 200 mV vs the lagtime (black lines). The plot in row i and column j represents the T_{ij} element of the matrix Data are compared with the Chapman-Kolmogorov predictions using a lagtime of either 10 (red symbols) or 1 ns (black symbols). Data refer to the reduced model with 7 states, transitions to/from the low probability states 5 and 6 are not shown.

It is evident that significant deviations of the markovian predictions from the true Ts are present at 1 ns lagtime, while the predictions for a lagtime of 10 ns are closer to the real behavior, although small differences can still be appreciated. A similar conclusion about the significant non-markovian behavior at 10 ns lagtime can also be drawn by looking at the implied time scales for the reduced MSM that are related to the probability matrix eigenvalues by the relationship:

$$t_i = -\frac{\Delta t}{\ln \lambda_i}$$

Where Δt is the lagtime used for the construction of the probability T and λ_i represent the module of the i-th eigenvalue. The implied timescales for a homogeneous MSM represent a kinetic property of the model that should not depend on the lagtime used for the construction of the probability T. For MD-derived MSMs plots of t_i vs Δt display a monotonically increasing behavior, with a stabilization at relatively large Δt. Thus, a method commonly used to establish the optimal lagtime is to look at the t_i vs Δt plot, and determine the lagtime as value where the implied timescales cease to increase (36, 37). Figure 10 reports the t_i vs Δt plot for the reduced MSM obtained in our case, showing that at 10 ns a nearly complete stabilization of the implied timescales is reached.

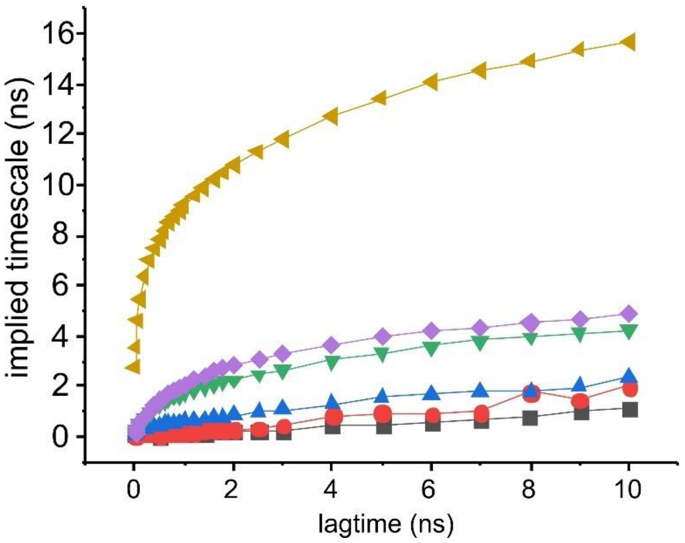

Figure S3. Implied timescales as a function of the lagtime for the reduced MSM model.

6. Comparison of the reduction procedure with flux matrices extracted from MD trajectories

Once the reduction strategy of the MSM has been defined, it is possible to assign each frame of the simulated MD trajectories to the macrostates of the reduced model. Therefore, the elements of the flux matrixes at any lagtime, $F_{ij}(n\Delta t)$, can be obtained directly from the MD trajectories by isolating all the transitions from state i to state j in the time interval nΔt, and counting the number of fractional conduction events in each of these trajectories. The element i,j of the flux matrix is then estimated as the average over all the trajectories connecting state i with state j in the time interval nΔt. The flux matrices calculated with this procedure at different lagtimes were compared with the corresponding flux matrices computed with the method based on equation 15. Figure S4 shows the average distance between the two estimates calculated as:

$$d(n\Delta t) = \frac{\sqrt{\sum_{ij}\left(F_{ij}(n\Delta t) - F_{ij}^{MD}(n\Delta t)\right)^2}}{NxN}$$

Where $F_{ij}(n\Delta t)$ and $F_{ij}^{MD}(n\Delta t)$ are respectively the flux matrices computed with equation 15 or estimated directly from simulated trajectories, and N is the number of states (7 in this case). The distance is zero at the shortest lagtime, which proves the correctness of the algorithm described in section 2 at this lagtime. At the longest lagimes considered, the distance between the two estimates of the flux matrix is around 0.015, in agreement with the observation that the flux matrix estimated using equation 15 reproduces the current observed in MD trajectories (Figure 9). Figure S5 reports the single elements of the flux matrices for transitions among the 5 states with significant probability of the reduced model estimated with the two methods. The predictions based on equation 15 agrees with estimates from MD trajectories.

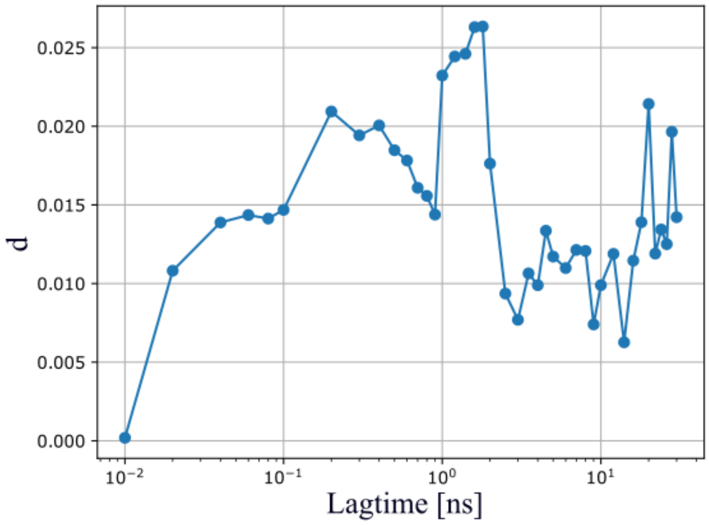

Figure S4. Distance between flux matrices estimated either using equation 15 or directly from MD trajectories.

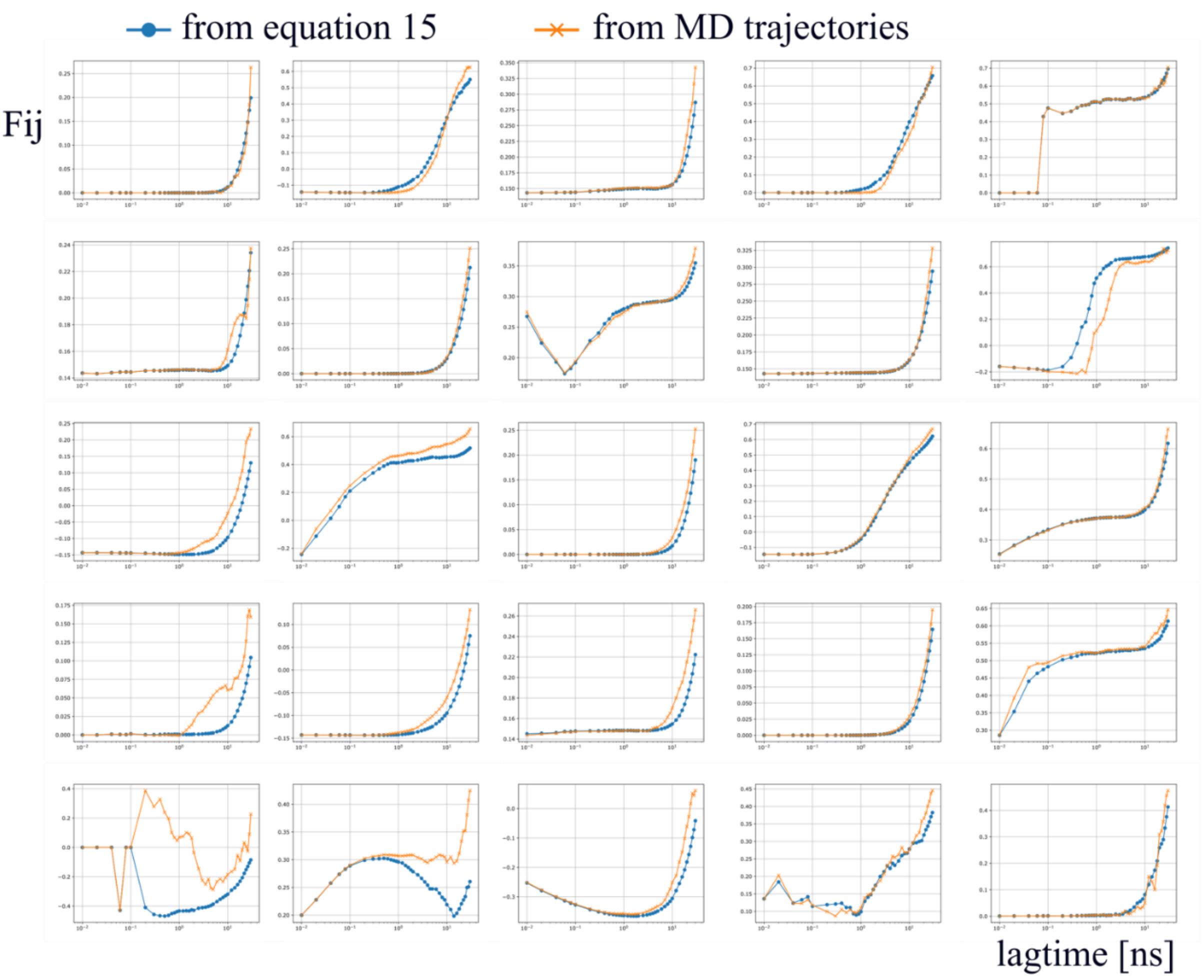

Figure S5. The plot in row i and column j represents the F_{ij} element of the flux matrices estimated respectively using equation 15 (blue lines) and from MD trajectories (orange lines). Data refer to the reduced model with 7 states, elements of the flux matrix for transitions to/from the low probability states 5 and 6 are not shown.
